# Dynamics and Applications of Photon-Nanostructured Systems

**DOI:** 10.3390/nano10091741

**Published:** 2020-09-03

**Authors:** Evangelia Sarantopoulou

**Affiliations:** Theoretical and Physical Chemistry Institute, National Hellenic Research Foundation, 48 Vassileos Constantinou Avenue, 11635 Athens, Greece; esarant@eie.gr; Tel.:+ 30-210-7273840

In a speedy and complicated word, only a small number of book readers have the time to dig out the hidden “gemstones” between the text lines. The ambition of the present Special Issue titled “Dynamics and Applications of Photon-Nanostructured Systems” is to offer the readers the opportunity to look at nanosystems differently. Besides photon surface engineering, topics, such as non-equilibrium nano thermodynamics, nonlinear dynamic evolution of nanosystems, quantum, size-effects, and photonic states from photon–nanosystem interactions are also discussed. Naturally, the description “photonic–nanostructured systems” outline photonic-crafted nanodevices with specific functionalities [1,2]. However, because electromagnetic waves are carriers of information, photons inscribe not only nanostructured domains in engineering materials but, also, frequency, phase, noise, and the state of quantum coherence are carriers of information of the structure and shape of nano-entities. The present Special Issue leverages the above topics and compiles twelve original theoretical and applied research articles and three review papers. The articles cover a broad range of thematic areas in physics, engineering, and biology, including photonic interaction with nanocavities and resonators. The collection of articles underlines not only a connection between light, nanodimensionality, surface, and dynamics, but it also accentuates essential issues such as topology, hierarchy, time, information, and irreversibility.

Contrary to common conceptions for time, light, surface, and dynamics, topology, hierarchy, information, and irreversibility are notions not readily discussed in regular nanotechnology research topics. However, these “hidden treasures” are in a position to shape tomorrow’s world by inspiring an implausible set of applications. Topology is the science of geometrical aspects of objects, aiming to identify geometrical invariants. In that sense topology and physics are inherently interconnecting because both scientific domains trace “*immutability*”. Time, on the other hand, in the classical conception of dynamics and quantum mechanics, flows continuously in a homogeneous way, following only a universal constraint that the equations of motion are invariant in time reversal. In this *static* and *reversible* world, dynamical systems are traced not only back, but their states are also projected in future. In the non-relativistic view, the physical observables, such as momentum, energy, and angular momentum, stand for the eternal, immutable landmarks of the physical–geometrical space. The topology is Euclidian, and the flow of time and the structure of space are both homogeneous. However, even in this immovable world, some hidden gems are waiting to be discovered. 

Let us clarify the above statement by giving an example. Two cars climb a mountain from opposite sides (Figure 1a). They both aim to reach the top of the hill. At first glance, the situation might well describe two similar processes. However, the two events might have diverging endings, and, therefore, the two events represent two different states. If the slopes and the morphology of the two hillsides are alike or even very similar, we expect the physical observables, associated with the two cars, to attain comparable values in both directions during the up-hilling stage. The two vehicles are in the position to reach the top of the hill because of a similar hillside topology. In the opposite case, where the slopes attain different values, the two vehicles require diverging energy, power, and torque to achieve the goal of reaching the top of the mountain. In the extreme case, where the inclination of one side exceeds, say, 50°, the associated vehicle might reach the top of the hill, but it might overturn because of the high speed required to climb the mountain (Figure 1b). We have here an example of instability in the “mountain–car” system during the dynamical evolution of the system imposed by the Euclidean topology. The excess power required to climb the steeper side of the mountain might be responsible for a car overturned at the top. 

The final state of the system “mountain–car” depends on the topology of the mountain surface. In the case of vehicle demolition during the climbing stage, ascending the top of a hill with a vehicle might be irreversibly dangerous, because topology might lead to an irreversible damaging state.

We have, here, a simplified example where surface topology entails different endings (states) of similar physical processes and also imply irreversibility during the evolution of a system in the same steps following classical dynamics. The two states and the topology imply a hidden degeneracy of physical observables, e.g., different values of vehicles’ power along opposite directions to obtain the objective of reaching the top of the mountain. With a similar way in 2D nanosystems, Euclidean topology is directly correlated with the topology of the electric components of a surface (local charge, electric field, potential). Because of hidden degeneracies and space inhomogeneity, we expect different physical phenomena to appear in tracing paths along opposite directions. It is plausible therefore to ask ourselves the question: “could one identify similar hidden degeneracies (‘hidden gems’) during light interaction with surfaces, and in the case of an affirmative answer, what kind of ‘gems’ one could be in a position of digging out?” Following the example of the two cars, we are in the position to conclude that irreversibility, time, and topology are connected inherently in the melting pot of classical notions of physics and because of everyday experience, humans realize that irreversibility is a rather ordinary state of Nature. The perceptions of evolution and irreversibility appear to be central in our understanding of the Cosmos and life. Irreversibility and evolution emerged at full speed during the nineteenth century in almost every scientific field and physics through the second law of thermodynamics, the important principle of the increase of entropy. In this classical view of the word, the second law of thermodynamics describes molecular disorder, and Boltzmann’s thermodynamic equilibrium corresponds to a state of maximum probability. However, in the physics of tiny systems, as well as in the evolution of life, entropy and irreversibility imply transformations to *higher levels of complexity* (hierarchical levels) and “information” which, contrary to the static view of the world (e.g., the planetary motions), follow unidirectional evolution pathways (the mortality is an irreversible evolution process). Let us clarify the above points with another physical example: irradiation of a polymeric surface with low-energy density photons in the visible region of the spectrum leads to the *reversible* fast dynamic transient response of electron and vibrational states. After some time, the system retains its original state with a similar hierarchical level before irradiation.

On the contrary, irradiation of the same system with vacuum ultraviolet photons (VUV, 110–180 nm) modifies the surface in *a non-reversible* way putting the system at a *higher hierarchical level*. The topologies of the surface prior and after irradiation are different. The two physical states have different fractal dimensionality and structure, and transition from the non-irradiated state to the radiative one is unidirectional (irreversible).

The entropy (and thus the transfer of information) of a system might follow non-thermodynamic pathways, and thus the transfer of physical information in tiny topological spaces could be chaotic. It is plausible therefore to ask another question: “how one could relate thermodynamic irreversibility with time (characteristic time of a central physical process), and hierarchy, or even to ask for the correlation between hierarchy and non-thermodynamic chaotic (random) motions, where the physical rates of systems follow completely random pathways”. The key to this answer is again topology. Indeed, confinement and escape of molecules from tiny spaces follow either thermodynamic or chaotic behavior. The size and topology of physical entities and space outline a set of time-space boundaries between thermodynamic equilibrium and chaotic motions, between physical laws and chaos, where physical rates vary randomly. This is the result of entropic variations from molecular confinement in tiny spaces, emerging from an irreversible surface restructuring at a high hierarchical level [3].

In the applications domain now, nanotechnologies trail diverging steps of innovative technological applications and the bet, in this case, is the successful integration of molecular functionalities with the macro-world [4]. Photons, besides their use in all practical aspects of modern life, convey a vast amount of *quantum information*, which, when joining nanosciences and nanotechnological tools, allow one to visualize new technological breakthroughs such as quantum computing (stages of higher hierarchical levels) [5].

Along the above lines, an extensive scientific and technological effort has been devoted to designing and integrating micro-nano sensors with improved (molecular) sensitivity, quick responses, high stabilities, and robustness in lab-on-a-chip devices. Among applications, mechanical nanosensors are detecting mechanical frequency variations, wave velocity, pressure, and strain [6]. Therefore, it would not be possible in this Special Issue to leave out nanomechanics and nanoresonators. The latter belongs to a new class of nanoelectromechanical systems enabling applications such as atomic and molecular sensing and separation, molecular transportation, high-frequency signal processing, and bioimaging [6]. Here, we have a *reversible state* of matter, but an *irreversible state of photons* which occupy a higher hierarchical quantum state after the interaction of photons with matter. Along the above lines, the mechanical and quantum characteristics of nanomechanical resonators coupled to a superconducting resonator were theoretically studied by Choi et al. [7] and thus is possible to predict phenomena that could lead to the development of novel technologies for quantum information processing.

Whispering-gallery-mode microresonator-based sensors with high local field intensities also configure novel platforms for enhancing the interactions between light and matter in both *reversible* (matter) and *non-reversible* (photons) states. Such states could bear low detection limits, down to a single molecule and nanoparticles. An “open” sensing configuration with the whispering-gallery-mode microresonator-based sensor, to monitor chemical reaction progress in the water droplet, is discussed and supported by a proof-of-a concept demonstration by Lu et al. [8]. This “open” configuration arrangement provides a real-time accuracy and sensitivity chemical/biochemical reaction kinetics platform.

Furthermore, optofluidic microcavity laser systems bear a wide span of potential applications in tunable single-mode on-chip lasers, biosensors in photobiology and photomedicine. For the first time in aqueous media, Guo et al. [9] investigated theoretically and experimentally single-frequency laser and mode splitting phenomena in optofluidic microdisk device that combines solid-state dye-doped polymer microdisks with a microfluidic channel device.

Guided mode resonance (GMR) structures allow obtaining complete bioreaction information. Zhou et al. [10] systematically presented a parametric analysis elucidating the influence of structural design factors (i.e., grating period and groove depth) at the nanoscale for “grating–waveguide” GMR sensors performance to achieve higher angular sensitivity and optimized wavelength figure of merit. By combining the analytical model and numerical simulations, higher performance sensors with lower detection limits in biosensing can be designed.

In the applications domain, nanostructures have attracted considerable research interest for their many advantages in photonic sensors applications. The response of the passive-type visible-blind ultraviolet photodetectors of ZnO nanorod, with different structure morphologies, was investigated by Khan et al. [11]. The fabricated ultraviolet sensors based on the ZnO NR-gated AlGaN/GaN high electron mobility transistor structure with nanoscale fin isolation demonstrate high-responsivity. Moreover, the sensing mechanism upon UV illumination was revealed.

Furthermore, porous Si–SiO_2_ UV microcavities with the thickness of a few tenths nm were also applied as filters by Jimenéz-Vivanco et al. [12] to modulate a broad responsivity photodetector with a detection range from 300 to 510 nm. The photodetectors had a broad, but porous Si–SiO_2_ UV microcavities improved the broad response silicon photodetector inside specific UV range of wavelengths, and they can be applied as UV-heated mirrors or UV bandpass filters.

In the *information* domain, numerical simulations were applied by Cao et al. [13] to prove that the electromagnetic field formed in the localized region of the mesoscale dielectric sphere can be modulated by introducing a nanohole structure at its shadow surface. Thus the authors were able to improve the spatial resolution of the information transfer up to λ/40, well beyond the stable immersion diffraction limit. This finding is essential for advancing the particle-lens-based super-resolution technologies, including sub-diffraction imaging, interferometry, surface fabrication, enhanced Raman scattering, and optical tweezer.

Purtov et al. [14] reported on the fabrication of defect-free arrays of pillars with diameters down to 184 nm. The two-dimensional photonic structures compared to theoretical predictions from Monte Carlo simulations and the optical reflectivities of the nanopillar gratings were analyzed by optical microscopy and verified by coupled-wave simulations. Ding et al. [15] reported on near and deep-subwavelength ripples on stainless-steel surfaces. A qualitative description based on the surface plasmon polariton modulated periodic Coulomb explosion is proposed for the interpretation of their formation mechanism. In the work of Wei et al. [16], a miniaturized nanowire laser with high end-facet reflection was realized by integrating an Ag grating between the nanowire and the substrate. The proposed nanowire laser with a lowered threshold and reduced dimensions is significant in on-chip information systems and networks.

Nano/micro-scale native random levels of roughness are responsible for the field enhancement effect unwanted in current electrical and optical systems. Τo design and optimize the networks, including the selection of materials, structures, and operating conditions, the plasmonic local energy enhancement effect around the metal surfaces, is required. Fukuoka et al. [17] investigated, numerically, the plasmonic enhancement of the electromagnetic field energy density at the sharp tips of nanoparticles or nanoscale surface levels of the roughness of hydrogen-absorbing transition metals, Pd, Ti, and Ni. Last but not least, three review articles are included in this particular issue. A brief review of enhancing the photoelectric performance of nanostructured semiconductor-based photodetectors is presenting by Ding et al. [18]. The authors give the latest research surface/interface engineering. The key factors and the challenges for improving nanostructured photodetectors are also pointed out.

A comprehensive review by Tian et al. [19] focuses on the synthetic methods and optoelectronic properties of inorganic boron-based nanostructures. Also, the optoelectronic behaviors of known inorganic boron-based nanostructures and future applications are presenting.

Finally, an extensive review of materials design strategies and performance of high sensitivity resists for extreme ultraviolet (EUV) lithography at 13.5 nm by Manouras and Argitis [20] is presented in this Special Issue. The last review article entails both fundamental information on the radiation-induced processes in this spectral region and a large number of new ideas targeting at the design of new highly sensitive and top-performing EUV resists.

All authors are confident that this current Special Issue entitled “Dynamics and Applications of Photon-Nanostructured Systems” will not only provide experts in the field but also curious readers with overarching insights into this complex and high cross-disciplinary field.

## Figures and Tables

**Figure 1:**
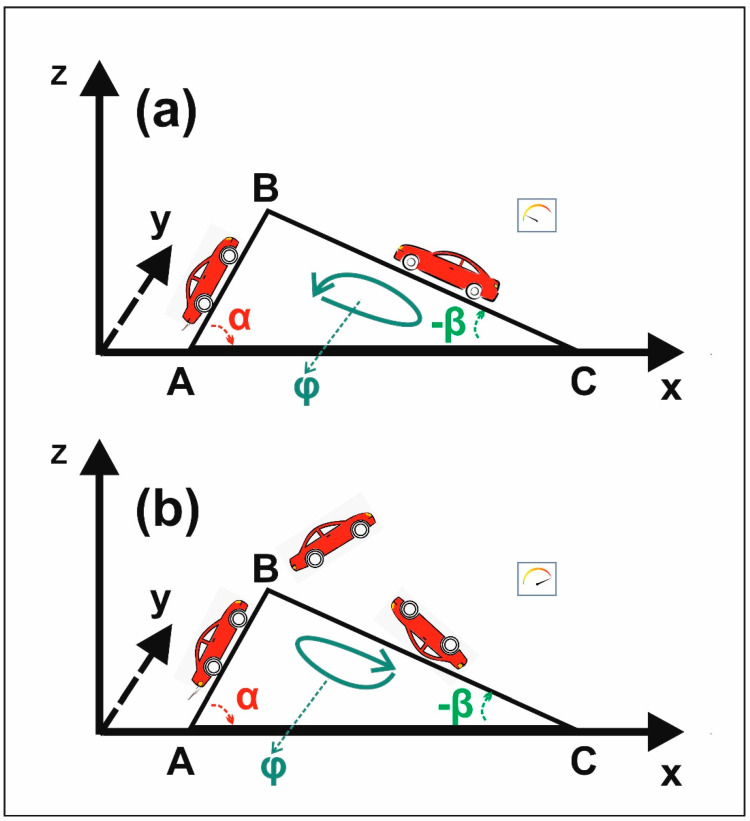
The connection between topology and irreversibility in classical dynamics: (**a**) Two cars climb two asymmetric sides of a mountain in opposite directions. Because of different side slopes, the engine power and the torque during the climbing stage are different; (**b**) the left car requires a high engine power and torque to reach the top of the mountain, which might lead to an over-flip (instability) and, thus, to an irreversible situation.
